# Coptis Root-Derived Hierarchical Carbon-Supported Ag Nanoparticles for Efficient and Recyclable Alkyne Halogenation

**DOI:** 10.3390/molecules30030567

**Published:** 2025-01-26

**Authors:** Cheng Liu, Fangyuan Gong, Xiaochuan Zou, Cun Wang, Zhengwei Xiong

**Affiliations:** 1 Chongqing Field Scientific Observation and Research Station for Authentic Traditional Chinese Medicine in the Tree Gorges Reservoir Area, Chongqing University of Education, Chongqing 400067, China; 2 College of Biological and Chemical Engineering, Chongqing University of Education, Chongqing 400067, China; gongfy@cque.edu.cn (F.G.); zxcvip2003@163.com (X.Z.); wangchun5224@126.com (C.W.)

**Keywords:** green chemistry, catalyst loading, biochar, silver nanoparticles (AgNPs), Coptis root, terminal alkyne halogenation reactions

## Abstract

The advancement of green chemistry and sustainable chemical processes has been significantly facilitated by catalytic systems derived from plant roots, which also present substantial application prospects in the realm of chemical synthesis. This study utilized the roots of Rhizoma Coptidis as a support to successfully fabricate a silver-based nanocatalyst. By depositing silver nanoparticles onto the root material of *Coptis chinensis* and subjecting it to carbonization, a silver/carbon composite was synthesized, featuring monodisperse silver nanoparticles and a hierarchical mesoporous carbon framework. This composite exhibits robust surface activity, a well-defined pore structure, and superior mechanical properties. The catalyst achieves a catalytic yield nearing 90%, showcasing remarkable activity in terminal alkyne halogenation reactions. Its stability and recyclability are markedly enhanced; it retains 95% of its mass and remains unaltered in the reaction solvent for over 160 h after five cycles. This method simplifies the synthesis of terminal alkynes and their derivatives, rendering the process more environmentally benign and efficacious. Furthermore, it broadens the potential applications of Rhizoma Coptidis in synthetic chemistry and pioneers a novel approach for the synthesis of precious metal catalysts from renewable resources.

## 1. Introduction

Rhizoma Coptidis root tissue is a natural, complex biomaterial composed of cellulose, hemicellulose, and lignin components. The main structural component of the Rhizoma Coptidis cell wall is cellulose, which provides the cell wall with microfibrous structural units that lead to good mechanical strength. In contrast, hemicellulose is a polymer consisting of many monosaccharides that can form a rigid fiber network while also promoting intercellular interlinking and structural integration. Lignin serves as a bonding and supporting factor in the cell wall, strengthening the cellulose structure. Lignin is an amorphous polymer consisting of methoxyphenylpropane substructures that is rich in functional groups (e.g., hydroxyl, carbonyl, and aldehyde). Therefore, lignin has distinct chemical properties and can be utilized as a reducing agent to prepare metal nanoparticles [1]. More importantly, the inherent three-dimensional interpenetrating network structure of lignin can serve as an ideal reaction platform for the production and stabilization of metal nanoparticles. For instance, the combination of lignin with silver nanoparticles (AgNPs) followed by carbonization can be employed to prepare silver/carbon (Ag/C) nanocomposites. These nanocomposites have remarkable properties due to their distinctive pore features and large specific surface area [2]. The alkaloids found in Rhizoma Coptidis root, including berberine, have aromatic and quaternary ammonium ring structures that may allow them to form stable complexes with metal ions, which would improve catalyst stability and dispersion [3,4]. In addition, Rhizoma Coptidis root is a common Chinese medicinal herb that is inexpensive and available from a variety of sources. Compared to typical metal oxide or carbon-based carriers, employing Rhizoma Coptidis root as a carrier can drastically lower catalyst preparation costs while providing greater economic benefits. Meanwhile, Rhizoma Coptidis root is a renewable plant resource with a short growth cycle that can be obtained indefinitely through both artificial and natural means. Using Rhizoma Coptidis root as a catalyst carrier is more sustainable than using nonrenewable metal mineral resources, which is consistent with green chemistry and sustainable development ideals. At the same time, Rhizoma Coptidis root waste is biodegradable and environmentally friendly. Using the multilayered, multidimensional, and multiscale structures generated by the long-term development of organisms in their natural environment as templates to prepare catalyst carriers offers a straightforward, green, and successful synthetic strategy for the construction of metal nanocatalytic systems [5,6,7,8]. However, there is currently little research into the synthesis of nanocomposites prepared using natural materials as both the reducing agent and carbon carrier precursor. It is possible that this high reactivity results from silver (I)’s exceptional π-Lewis acidity and carbon affinity, which allow it to form silver-π complexes and selectively activate carbon–carbon triple bonds [9].

AgNPs are among the most widely investigated nanomaterials in the field of metal nanoparticle research because of their distinct physicochemical characteristics [10,11,12]. Silver (I) has empty d orbitals that can easily interact with alkyne carbon–carbon triple bonds to form complexes. Therefore, due to their ability to effectively promote alkyne functionalization reactions, AgNPs are one of the best catalysts for alkyne hydrocarbon activation. Among the alkynes used in these reactions, a family of organic compounds known as terminal alkynes and their derivatives has at least one carbon–carbon triple bond (C≡C) and at least one triple-bonded carbon atom at the end of the molecule. This distinct molecular structure provides terminal alkynes with high reactivity, making them attractive precursors for the synthesis of high-value compounds [13]. For instance, these compounds are frequently employed to synthesize medicines, functional molecules, sensors, semiconductors, liquid crystals, and other materials and polymers. Moreover, terminal alkynes have vital applications in material science, biology, medicine, biotechnology, and nanotechnology [14,15]. Therefore, the conversion and utilization of terminal alkynes is a popular research hotspot. The Ag/g-C_3_N_4_ catalyst system reported by Shi et al. [16], which was prepared using AgNPs, is one example of a recent research advancement. This catalyst system showed outstanding catalytic performance in the halogenation reactions (i.e., the iodination, bromination, and chlorination) of terminal alkynes. Furthermore, N-heterocyclic carbene (N-heterocyclic carbene)-Ag nanocomposite catalysts were successfully prepared by Yu et al. [17]. These catalysts demonstrated exceptional catalytic activity in the carboxylation reaction of terminal alkynes with carbon dioxide at room temperature, and they also displayed exceptional chemical stability and reusability.

Nevertheless, there are still certain obstacles these catalysts must overcome in real-world applications. First, the catalytic efficacy and stability of AgNPs are significantly impacted by their size and dispersion. Smaller particles have a bigger specific surface area and more active sites, resulting in superior catalytic performance than larger particles. However, AgNPs with smaller sizes have higher surface energies, which may result in interparticle aggregation. This reduces the specific surface area, conceals the active sites, and weakens the catalytic performance of AgNP-based catalysts [18]. Therefore, a substrate or carrier is used to support the catalytically active components in these catalysts, which increases the number of active sites and improves their catalytic efficiency. At the same time, a carrier protects a catalyst from high temperatures or harsh chemical environments, meaning that loaded catalysts have better thermal and chemical stability [19,20,21]. Researchers have investigated a variety of carrier materials to effectively limit particle migration and aggregation while maintaining good dispersion via strong interactions between AgNPs and carrier materials, including porous carbon, iron oxide, silica, and alumina [2,22]. However, these carrier materials are difficult to prepare, and their stability and cyclic regeneration properties still need to be improved. Second, the methods used to prepare AgNPs have limits. Chemical reduction, UV reduction, and thermal degradation are currently the most widely used synthesis techniques for obtaining AgNPs [12,23,24,25]. Chemical reduction approaches are widely preferred due to their gentle conditions and ease of usage. However, the utilization of chemical reducing agents such as sodium borohydride or hydrazine hydrate could have negative environmental impacts. An additional pressing technical challenge associated with chemical reduction strategies is the efficient and comprehensive separation of AgNPs from these reducing agents at the next processing stage, which is necessary to remove any possible dangers to the environment and human health [12,26]. These problems restrict the broad use of AgNPs and make their production process more complicated.

In summary, even though silver nanocatalysts exhibit notable benefits in alkyne activation reactions, sustainable synthesis techniques still need to be developed to prepare these catalysts, and the efficient loading of active nanoparticles with high catalytic activity and long-term stability remains a major research concern. In this work, the unique structure of Rhizoma Coptidis roots was utilized to create a biochar catalyst carrier with a mesoporous structure. Due to its exceptional porosity, thermal stability, and surface functionality, this biochar carrier had ideal properties for supporting and immobilizing silver nanocatalysts. Using cells from the upper region of Rhizoma Coptidis (also referred to as the “bridge”) as a template and a silver–ammonia (Ag(NH_3_)_2_NO_3_) solution as a metal precursor, nanosilver/carbon (Ag/C) composite catalysts loaded with varying concentrations of AgNPs were prepared through an in situ reduction–pyrolysis process. The prepared Ag/C catalysts underwent structural investigation and thorough characterization. Finally, the prepared catalysts were utilized for the chlorination, bromination, and iodination of terminal alkynes. According to the experimental results, these silver nanocatalysts demonstrated outstanding catalytic efficiency (catalytic yield near 90%), good stability (unaltered performance within 160 h), and a high recovery of 95% after more than five cycles in the terminal alkynyl halogenation reactions.

## 2. Results and Discussion

### 2.1. Preparation of Silver Nanocatalysts Using Rhizoma Coptidis Root as a Template

**Figure 1:**
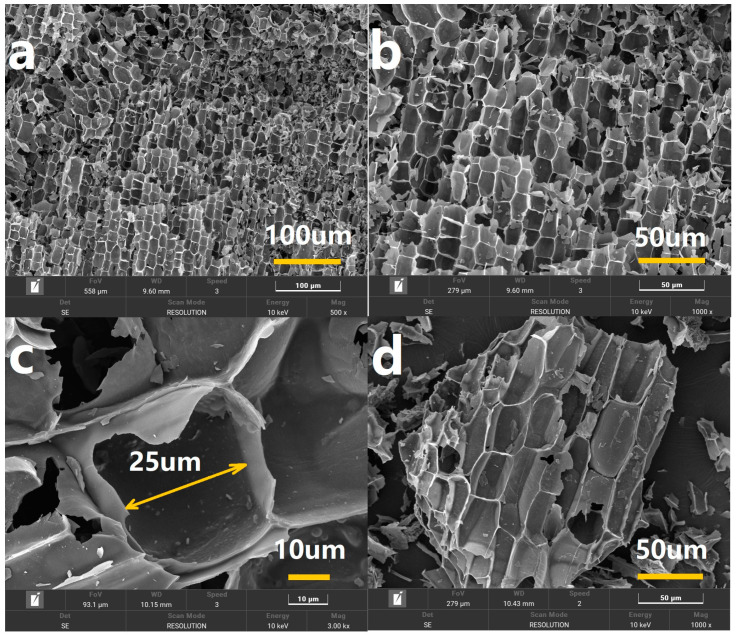
SEM images showing morphology of catalyst support prepared using blank Rhizoma Coptidis root template (**a**–**c**) before and (**d**) after grinding.

First, an unloaded Rhizoma Coptidis blank template was carbonized, and its surface shape was carefully examined to evaluate its potential as a catalyst carrier. Scanning electron microscopy (SEM) images were obtained at magnifications of 500× (Figure 1a), 1000× (Figure 1b), and 3000× (Figure 1c). These SEM images clearly showed the existence of a highly organized pore network created by the high-temperature carbonization of the Rhizoma Coptidis roots. With a consistent pore diameter of roughly 25 μm, the pores in the cell walls of the Rhizoma Coptidis roots were regularly aligned. This well-organized pore structure was expected to be highly suitable for the preparation of nanoparticles. Moreover, the dense pore network would provide a larger contact area between the catalyst and reaction solution, which was expected to boost catalytic efficiency. Next, the carbonized carrier was ground into smaller particles, as shown in the SEM image in Figure 1d. The porous lattice-like structure of the carrier was successfully preserved after grinding, indicating sufficient mechanical strength to act as a catalyst carrier. These findings suggest that Rhizoma Coptidis roots show excellent potential for use as a natural biomaterial in the production of catalyst carriers. This investigation establishes the groundwork for the subsequent preparation of nanosilver/carbon composite catalysts and their application in catalyzing the halogenation reactions of terminal alkynes.

**Figure 2:**
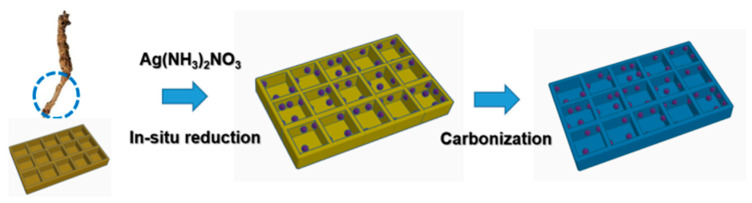
Schematic diagram of the preparation of nanosilver/carbon composite catalysts with Rhizoma Coptidis root as the carrier.

A wet impregnation strategy was utilized to load the Rhizoma Coptidis root carrier with AgNPs (Figure 2) [26]. The “bridge” portion of the Rhizoma Coptidis root was selected as the catalyst carrier template. These carrier particles were combined with silver–ammonia (Ag(NH_3_)_2_NO_3_) solution and reacted for three days under gentle stirring. During this reaction, the oxygen-containing functional groups in the Rhizoma Coptidis roots served as reducing agents during the synthesis process, where they aided the formation of monodisperse AgNPs on the plant surface [2]. The hue of the solution gradually turned yellowish brown, indicating the production of AgNPs. The generated product was then carbonized at a high temperature in the presence of nitrogen to transform the catalyst carrier into a biomass char with an embedded mesoporous structure. The “bridge” portion of the Rhizoma Coptidis root may have efficiently bonded with the AgNPs due to its high cellulose concentration, solving the problem of unevenly dispersed and easily aggregated AgNPs in the solution [2]. Furthermore, the “bridge” portion of the root had a thicker cell wall, which strengthened the carbonized lattice structure and provided the AgNPs with a sturdy, three-dimensional support structure. This was expected to improve the stability of the prepared Ag/C catalysts.

A series of catalysts with different silver loadings was prepared using (Ag(NH_3_)_2_NO_3_ solutions with different concentrations. These catalysts were denoted AG-1/C (0.02 M), AG-2/C (0.05 M), and AG-3/C (0.10 M). A blank carrier not loaded with AgNPs was also prepared, denoted AG-0/C. These catalysts were thoroughly characterized and then evaluated for the catalytic halogenation of alkynes.

### 2.2. Results of Catalyst Characterization

#### 2.2.1. N_2_ Adsorption–Desorption

**Figure 3:**
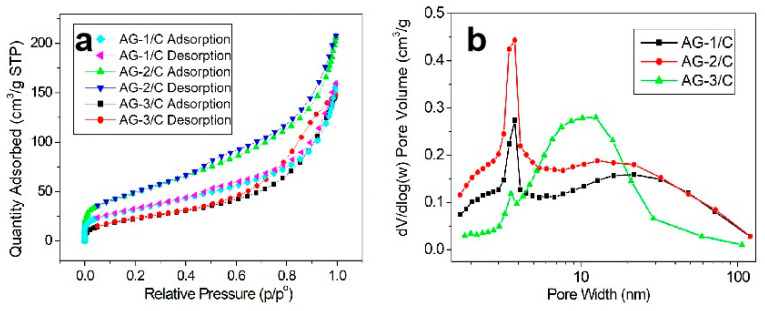
(**a**) N_2_ adsorption–desorption isotherms; (**b**) porosimetry profiles of the AG-1/C, AG-2/C, and AG-3/C catalyst.

The specific surface areas and pore structure characteristics of the AG-1/C, AG-2/C, and AG-3/C catalyst samples are displayed in Table 1. The N_2_ adsorption–desorption isotherms and the porosimetry profiles are shown in Figure 3. Figure 3a illustrates the N_2_ adsorption and desorption isotherms for the three catalyst samples. The isotherms demonstrate typical Type IV behavior, including a hysteresis loop, which is characteristic of mesoporous materials. As the concentration of the Ag(NH_3_)_2_NO_3_ solution increased, a notable increase in specific surface area was observed. Moreover, the average pore diameter of Ag-3/C (7.26 nm) was 31.6% smaller than that of AG-1/C (10.61 nm). This suggests that the Ag loading increased with increasing solution concentration, providing more active sites for the catalytic reaction. The pore volume of AG-3/C (0.32 cm^3^/g) was 44.5% larger than that of AG-1/C (0.22 cm^3^/g), as depicted by the integrated pore volume under the pore size distribution curve in Figure 3b. The greater degree of porosity in Ag-3/C was expected to enhance the diffusion efficiency of reactants and improve the stability and longevity of this catalyst.

#### 2.2.2. XRD Analysis

**Figure 4:**
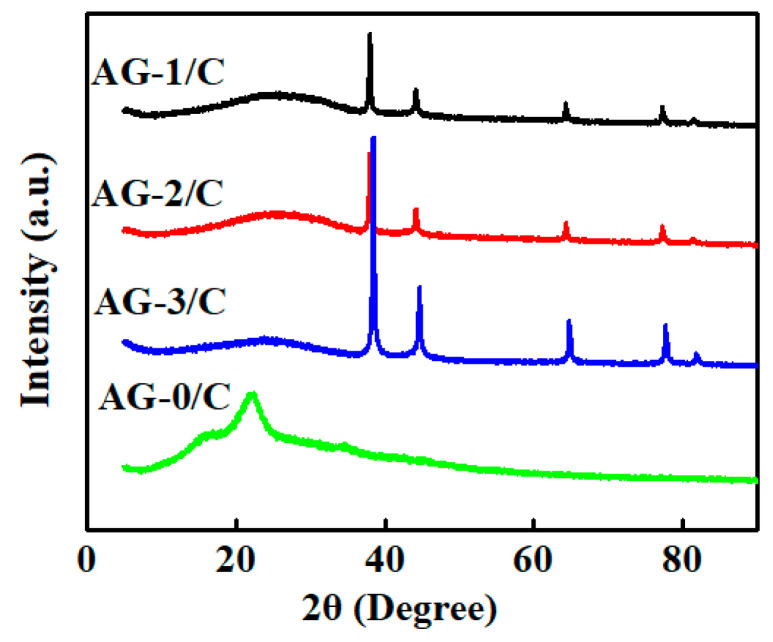
XRD patterns of Ag/C catalysts.

The X-ray diffraction (XRD) patterns of the synthesized Ag/C catalysts are shown in Figure 4. According to these diffraction patterns, the loaded silver in the catalysts was present in the form of AgNPs, and the patterns varied with the dosage of Ag(NH_3_)_2_NO_3_ solution.

The XRD pattern of the blank carrier (AG-0/C), which was not treated with silver–ammonia solution, did not show significant diffraction peaks. This indicated the absence of AgNPs on this carrier. In contrast, the XRD patterns of AG-1/C, AG-2/C, and AG-3/C showed characteristic diffraction peaks at 2θ values of 38.4°, 44.2°, 64.6°, and 77.3°. These diffraction peaks, respectively, corresponded to the (111), (200), (220), and (311) crystal planes of Ag, confirming that the silver in these three catalysts mainly existed as zero-valent metallic silver [27]. Moreover, the diffraction peak intensities increased with increasing silver–ammonia solution concentration. This phenomenon was attributed to an increase in the size of the AgNPs or the higher proportion of silver in the samples prepared with higher solution concentrations [18,20]. Importantly, larger AgNPs or a higher silver content can provide more active sites, leading to enhanced catalytic activity.

#### 2.2.3. FT-IR Analysis

**Figure 5:**
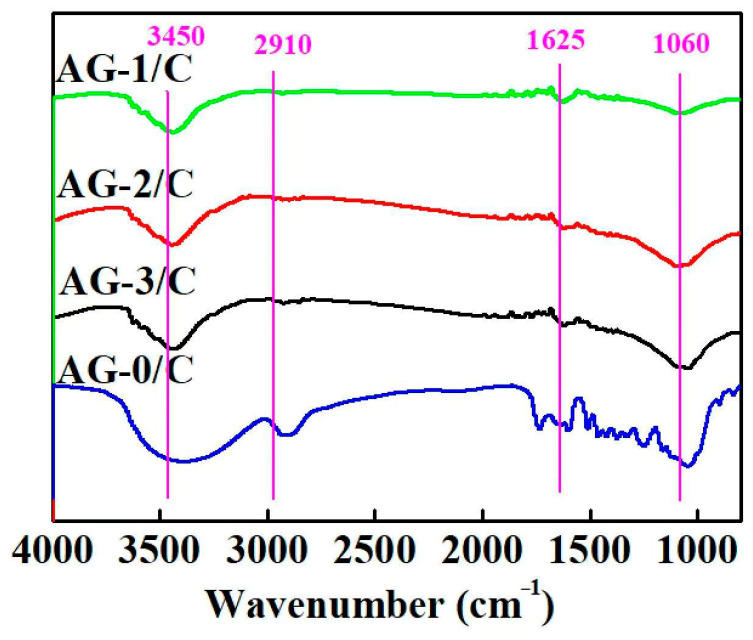
FT-IR spectra of AG/C catalysts.

The Fourier transform infrared (FT-IR) spectra of the blank carrier (AG-0/C) and the three silver-loaded catalysts (AG-1/C, AG-2/C, and AG-3/C) are presented in Figure 5.

In the FT-IR spectra of all of the four catalyst samples, the corresponding characteristic peaks can be observed. The absorption bands between 3450 and 2910 cm^−1^ were attributed to the stretching vibration of the O-H bond and the asymmetric stretching vibration of the C-H group, respectively [28]. Furthermore, the peaks at 1625 cm^−1^ and 1060 cm^−1^ were the typical absorption bands for cellulose and lignin, corresponding to the stretching vibration of C=C and C-O-C bonds, respectively [29]. These distinctive absorption peaks confirmed the presence of a large number of polar, oxygen-containing functional groups on the carrier surface that could be utilized as important AgNP anchoring sites. Therefore, in addition to aiding in the reduction in the AgNPs, the oxygen-containing functional groups in the Rhizoma Coptidis root structure improved the interaction between the AgNPs and carrier surface via chemisorption, potentially increasing the structural stability and catalytic activity of the composite catalysts. Consequently, the steady loading of AgNPs was dependent on these functional groups.

#### 2.2.4. TGA

**Figure 6:**
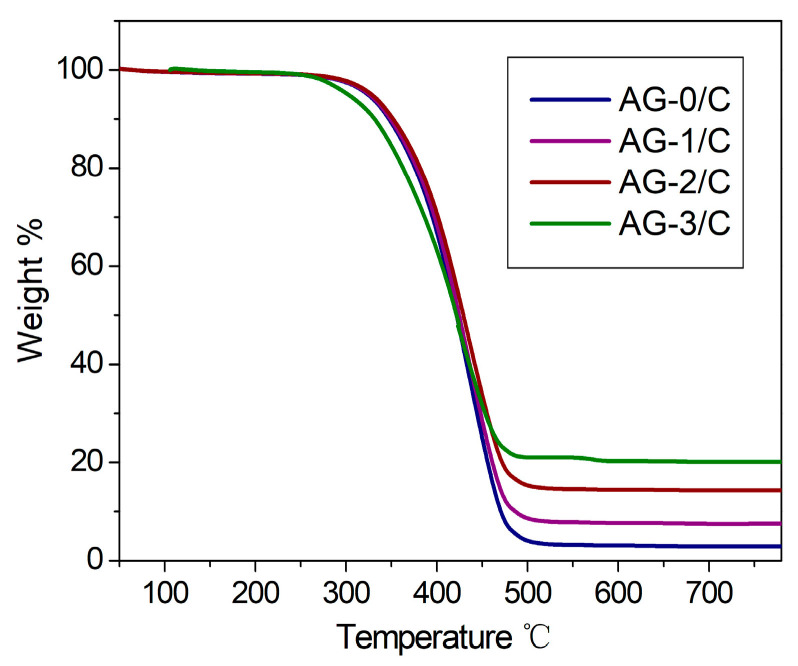
TGA curves of Ag/C catalysts.

Thermogravimetric analysis (TGA) was employed to quantify the loading of AgNPs in the three silver-loaded catalysts, as shown in Figure 6. At temperatures lower than 300 °C, weight loss was ascribed to the evaporation of physically adsorbed water from the catalysts. Within the temperature range of 300–500 °C, weight loss was mainly due to the thermally induced breakdown of the organic carbon skeleton. After the TGA, AG-1/C, AG-2/C, and AG-3/C had weight residuals of 7.5, 14.3, and 18.7 wt%, respectively. These results are in line with the different concentrations of Ag(NH_3_)_2_NO_3_ solution used to prepare the catalysts and correlate to the amount of metallic silver present in each catalyst. Therefore, the loading of AgNPs in the catalysts was successfully regulated by varying the initial concentration of the silver–ammonia solution.

#### 2.2.5. SEM and TEM Analysis

**Figure 7:**
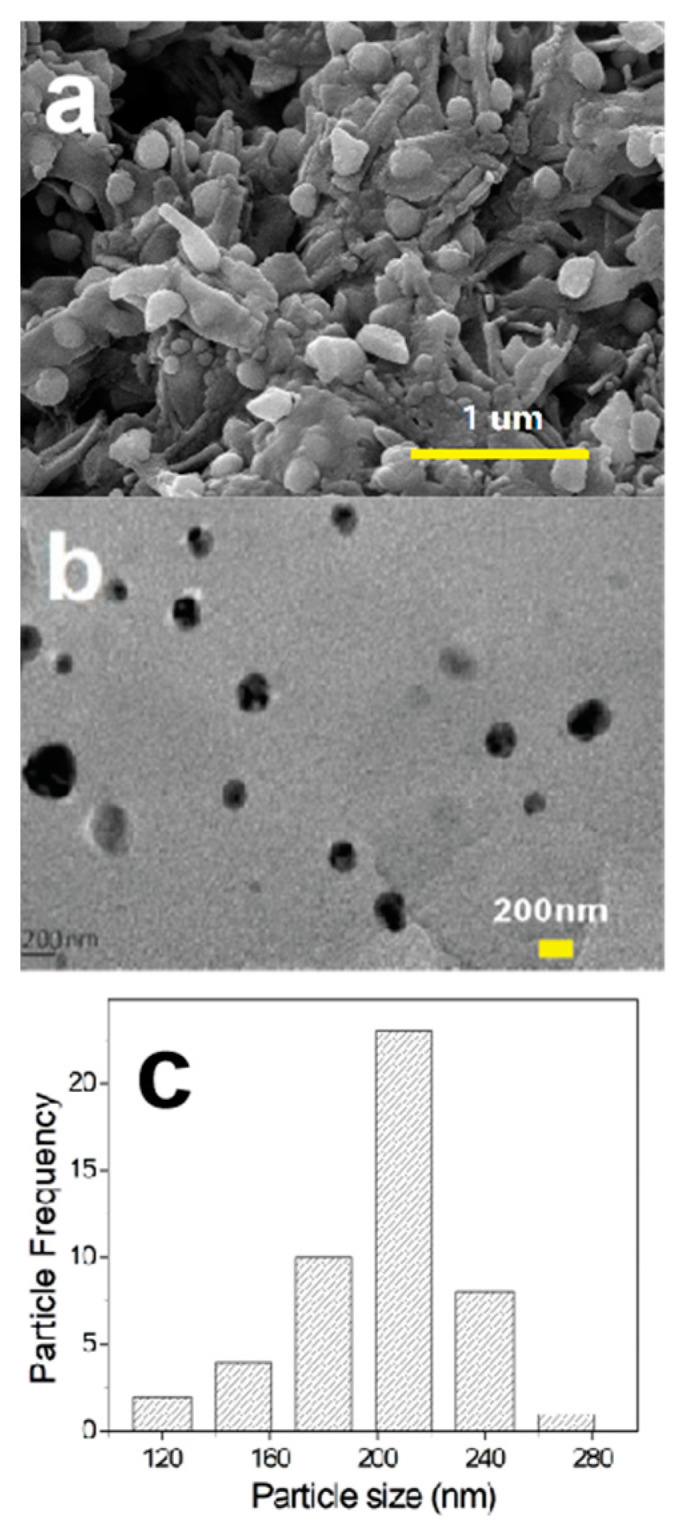
(**a**) SEM image showing surface morphology of AG-3/C catalyst, (**b**) TEM image displaying AgNPs on AG-3/C, and (**c**) particle size distribution of AgNPs on AG-3/C.

The surface morphology and the distribution of metallic AgNPs in the AG-3/C catalyst were evaluated by scanning electron microscopy (SEM) and transmission electron microscopy (TEM), as shown in Figure 7.

The AgNPs firmly adhered to the biomass charcoal template provided by the Rhizoma Coptidis roots (Figure 7a). The reticular structure of the template was composed of thick cell walls, which demonstrated exceptional mechanical resilience by remaining intact and not collapsing, even after being loaded with AgNPs. After grinding, the surface morphology of this catalyst was further evaluated by TEM, which showed a consistent dispersion of AgNPs on the carrier (Figure 7b). These particles had a uniform size distribution and a regular spherical shape with distinct edges. The sizes of 50 randomly chosen particles were measured, and according to the resulting particle size distribution (Figure 7c), the particles were primarily concentrated in the 120–260 nm range. This finding suggests that by evenly dispersing AgNPs on the biomass char carrier produced using Rhizoma Coptidis roots and taking advantage of the stabilizing effect of the carrier on the silver particles, AgNP agglomeration was successfully suppressed and particle size uniformity was maintained [2,22]. Electroplating technology is a significant approach for creating composite materials because it allows for the creation of homogenous and dense composite coatings, as well as easy control over coating thickness and composition [30]. However, during the electroplating process, nanoscale or micrometer-level dopants (such as graphene) tend to agglomerate in the plating solution, resulting in flaws in the composite coating [31]. In contrast, employing Rhizoma Coptidis root as a carrier to create silver nanocatalysts can effectively address this issue. Furthermore, the Rhizoma Coptidis root carrier approach outperforms electroplating technology in terms of biocompatibility, renewability, preparation cost, and adherence to green chemistry principles. Notably, the AgNPs and the Rhizoma Coptidis root cells were very tightly bonded. Even after carbonization for 2 h at 700 °C and grinding, the AgNPs remained monodisperse (Figure 7b). This was ascribed to the mesoporous structure of the Rhizoma Coptidis roots, which was generated during the carbonization process. This structure enhanced the stability and dispersion of the AgNPs, in addition to providing a stable support. Overall, the structure and morphology of this catalyst provided a large number of active sites and superior mass transfer performance, showing excellent promise for achieving improved catalytic performance [2].

#### 2.2.6. XPS Analysis

**Figure 8:**
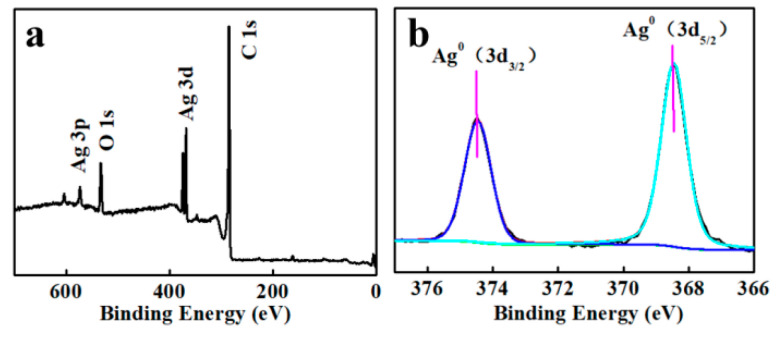
XPS (**a**) survey and (**b**) Ag 3d spectra of AG-3/C catalyst.

The chemical valence states of the components in the AG-3/C catalyst were characterized using X-ray photoelectron spectroscopy (XPS). The survey spectrum of AG-3/C showed four prominent peaks at 573.1, 531.4, 372.5–367.1, and 285.0 eV that were ascribed to Ag 3p, O 1s, Ag 3d, and C 1s, as shown in Figure 8a. In addition to elemental carbon and silver, AG-3/C also contained a notable oxygen concentration, in agreement with the FT-IR analysis. The Ag 3d region was further analyzed, as shown in Figure 8b. Two significant peaks near 368.5 eV and 374.5 eV corresponded to the binding energies of the 3d 5/2 and 3d 3/2 orbitals of Ag^0^, respectively. This indicates that the silver in the sample mainly existed in the form of zero-valent metallic AgNPs [32], in agreement with the XRD analysis.

### 2.3. Results of Evaluation of Catalytic Performance

**Figure 9:**
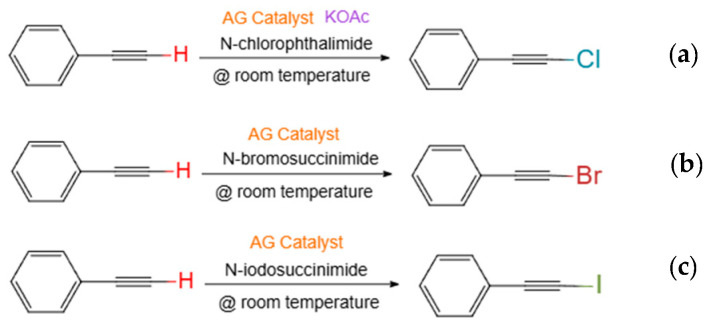
(**a**) Chlorination, (**b**) bromination, and (**c**) iodination of phenylacetylene catalyzed by Ag/C catalyst.

The performance of the AG-1/C, AG-2/C, and AG-3/C catalysts was evaluated using phenylacetylene as the reaction substrate. To study the performance of the catalysts for terminal alkyne halogenation reactions (chlorination, bromination, and iodination), the substrate was reacted with N-chlorophthalimide (NCS), N-bromosuccini-mide (NBS), and N-iodosuccinimide (NIS), as shown in Figure 9. All reactions were performed at room temperature for 6 h using a 20 mg catalyst dosage. Following the completion of the reaction, ethyl acetate was added to the reaction solution. Gas chromatography–mass spectrometry (GC-MS) was then utilized to evaluate the effectiveness of each catalyst, as shown in Table 2. The GC profiles for the halogenation reactions catalyzed by AG-1/C, AG-2/C, and AG-3/C are presented in the Supplementary Information Figure S1.

Remarkably, all three catalysts demonstrated effective catalytic activity for the halogenation of phenylacetylene as a representative terminal alkyne. After the reaction, the phenylacetylene signals vanished, and all groups achieved 100% conversion. The average catalytic yield of AG-1/C was almost 60%, while the AG-2/C and AG-3/C catalysts achieved yields of nearly 90%. This variation was directly correlated with the AgNP content of the catalysts. AG-1/C may have contained more biochar due to its lower metal loading, and some of the polar functional groups of the biochar may have caused adverse reactions, impacting the effectiveness of this catalyst [33]. Of the three catalysts, AG-3/C showed the best performance in all three reactions.

### 2.4. Results of Catalyst Recycling

The recoverability of a non-homogeneous catalyst directly affects its economic viability and environmental sustainability [34,35]. Therefore, the recyclability of the AG-3/C catalyst was evaluated, and the recovered catalyst was characterized. At the end of each reaction cycle, this catalyst was cleaned and then calcined to restore its catalytic activity for use in subsequent reaction cycles. This ensured the purity of the recovered catalyst and prevented organic residues from affecting catalytic performance.

**Figure 10:**
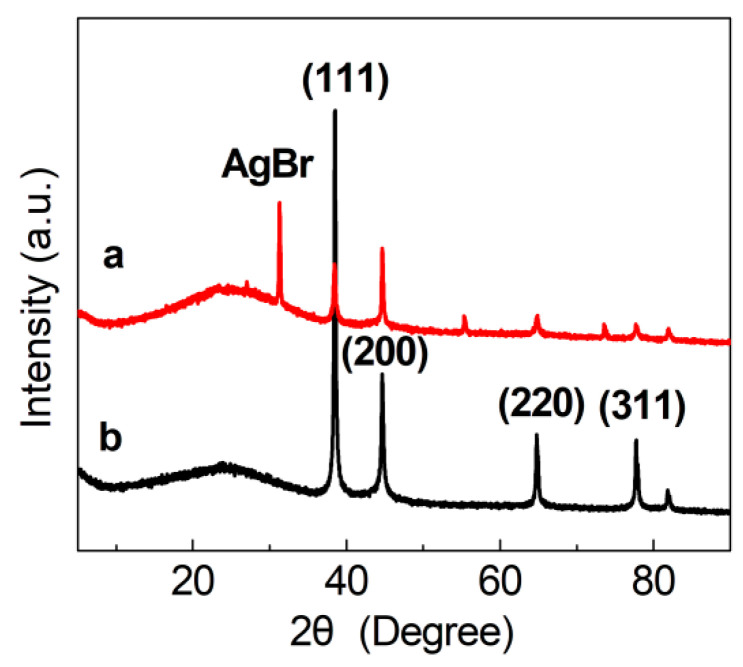
XRD patterns of AG-3/C catalyst (**b**) before and (**a**) after the bromination of phenylacetylene.

The AG-3/C catalyst was directly recovered after the bromination reaction for evaluation via XRD, as shown in Figure 10. A comparison of the XRD patterns obtained before (Figure 10b) and after (Figure 10a) the reaction showed that the main structure of the catalyst remained unchanged. However, the post-reaction XRD pattern showed small silver bromide diffraction peaks, which could be attributed to the slight oxidation of the AgNPs on the catalyst surface during the reaction or post-treatment process. However, these AgNPs can be efficiently reduced back to their original zero-valent metallic state by further treatment.

**Figure 11:**
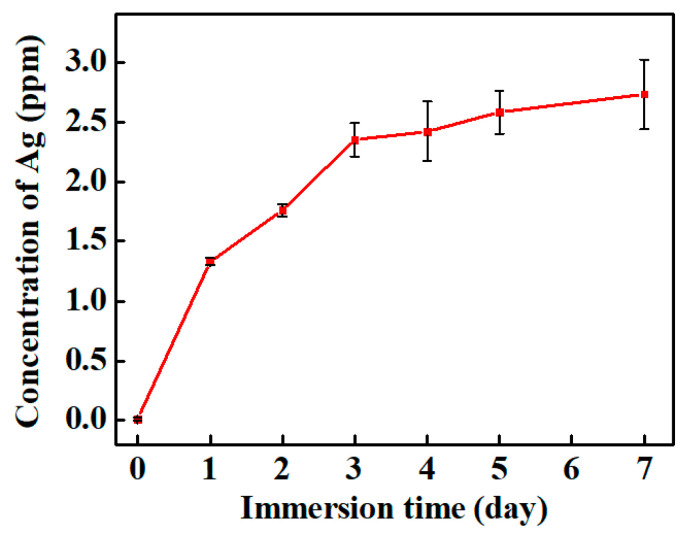
Silver leaching test results for AG-3/C catalyst.

An accelerated-aging test method was employed to examine the long-term stability of the AG-3/C catalyst. In this test, AG-3/C was slowly and continuously stirred in acetonitrile, which was the solvent employed for the catalytic reaction. An inductively coupled plasma (ICP) technique was utilized to precisely measure the elemental silver concentration leached into the immersion solution, and the outcomes are displayed in Figure 11. After the 168 h (7-day) immersion process, the elemental silver concentration was still less than 3 ppm, unequivocally confirming the incredibly strong bond between the AgNPs and the Rhizoma Coptidis root-based carrier. This robust bonding guarantees the durability of the catalyst at the macroscopic level and demonstrates the potential for utilizing nanosilver/carbon catalysts in industrial applications, particularly those that require frequent or prolonged use.

Traditional powder catalysts can undergo fragmentation due to thermal or mechanical shocks in actual applications, resulting in catalytic attrition and deactivation [36]. Furthermore, traditional powder catalysts typically have low thermal conductivity, which can lead to the formation of hot spots during intense exothermic reactions. This can compromise the stability and activity of catalyst materials [37]. Moreover, the insufficient chemical stability of many powder catalysts means that their overall performance can decline over time [38]. All of these factors lead to catalyst deactivation, meaning that traditional powder catalysts may require regular replacement and regeneration. Furthermore, the recycling and reuse of typical powder catalysts are challenging from a technical and financial standpoint because of abrasion and deactivation, particularly if the catalyst particles break or the active ingredients are lost [39,40].

**Figure 12:**
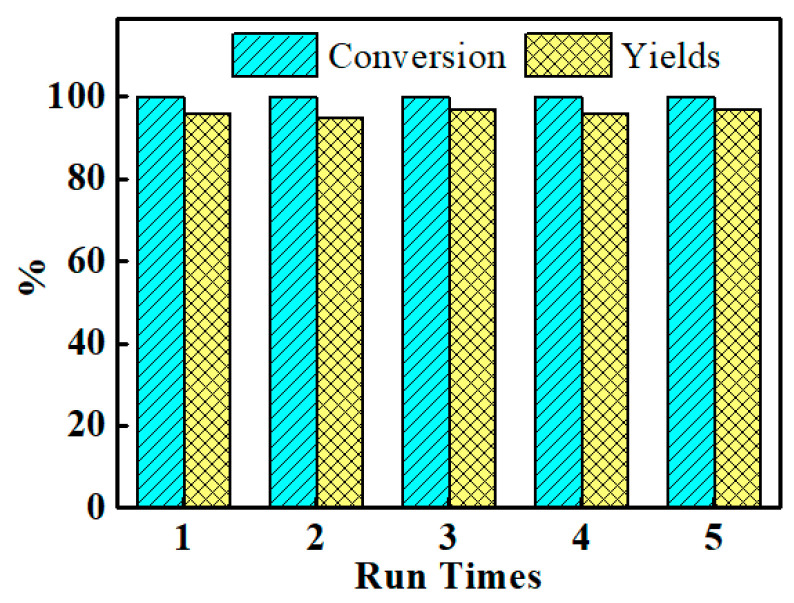
Cycling test results of AG-3/C catalyst for the bromination of phenylacetylene.

In this work, the performance of AG-3/C was evaluated in five successive catalytic bromination cycles to thoroughly assess its recycling capacity, as shown in Figure 12. According to the experimental data, the AG-3/C catalyst demonstrated exceptional catalytic activity and stability in each cycle. The catalytic yield exceeded 95% and the reaction conversion reached 100% even in the fifth cycle, with no discernible declining trend. Compared with other Ag catalysts (AgNO_3_ catalytic conversion rate of 78%, yield of 54%; AgSO_4_ catalytic conversion rate of 65%, yield of 47%; and Ag_2_O catalytic conversion rate of 92%, yield of 76%) [41], the Rhizoma Coptidis root-supported catalyst has significant advantages in the conversion rate and yield of terminal alkyne halogenation reactions, which may be attributed to the synergistic catalytic effect between alkaloids and silver nanoparticles in Rhizoma Coptidis root, promoting electro-transfer and the generation of reaction intermediates [3,4]. Moreover, the mass retention of this catalyst after five cycles was 95.4%. This result confirmed the exceptional stability of this catalyst and demonstrated its notable advantages compared to traditional powder catalysts. These advantages were primarily ascribed to the strong interactions between the AgNPs and the carrier as well as the excellent mechanical strength of the carrier structure [42,43].

## 3. Materials and Methods

### 3.1. Sampling of Catalyst Carriers from Rhizoma Coptidis Roots

Rhizoma Coptidis roots were washed with water to remove the clustered dirt, fibrous roots, and contaminants. Next, the washed roots were dried in an oven for 5–6 h at 50 °C. After drying, the “bridge” portion of the roots was cut into 2–3 mm granules for further use. This section included roots with a smooth surface and a length greater than 20 mm.

### 3.2. Metal Catalyst Loading

An ammoniacal silver nitrate (Ag(NH_3_)_2_NO_3_) solution was prepared by dropping ammonia (10% mass fraction) into silver nitrate (AgNO_3_) until the solution turned transparent. Three different Ag/C catalysts were prepared by immersing 3 g of the Rhizoma Coptidis root granules in 50 mL Ag(NH_3_)_2_NO_3_ solutions with different concentrations of Ag(NH_3_)_2_NO_3_ (0.02, 0.05, and 0.1 M) at room temperature. Each mixture was slowly stirred for three days until the yellowish brown hue of the Rhizoma Coptidis root granules stabilized.

### 3.3. Catalyst Preparation with Biomass Carbon Template Loading

After immersion for three days, each sample was removed from the Ag(NH_3_)_2_NO_3_ solution and washed with deionized water to remove the surface solution, followed by natural drying at room temperature. Each sample was then carbonized under nitrogen protection at 700 °C for 2 h, which caused the sample color to become black.

### 3.4. Verification of Catalytic Effect of Halogenated Reaction

Phenylacetylene was utilized in this work to evaluate the performance of the catalysts for the halogenation of terminal alkynes.

Chlorination reaction: Phenylacetylene (0.3 mmol, 30.6 mg), NCS (0.45 mmol), potassium acetate (0.3 mmol, 29.4 mg), 20 mg of the prepared catalyst, and 3 mL of acetonitrile were added to a 10 mL brown flask and stirred at room temperature for 6 h. When the reaction was complete, the reaction mixture was diluted with ethyl acetate and then evaluated using gas chromatography–mass spectroscopy (GC-MS).

Bromination reaction: Phenylacetylene (0.3 mmol, 30.6 mg), NBS (0.45 mmol), 20 mg of the prepared catalyst, and 3 mL of acetonitrile were added to a 10 mL brown flask and stirred at room temperature for 6 h. When the reaction was complete, the reaction mixture was diluted with ethyl acetate and evaluated using GC-MS.

Iodination reaction: Phenylacetylene (0.3 mmol, 30.6 mg), NIS (0.45 mmol), 20 mg of the prepared catalyst, and 3 mL of acetonitrile were added to a 10 mL brown flask and stirred at room temperature for 6 h. When the reaction was complete, the reaction mixture was diluted with ethyl acetate and then evaluated using GC-MS.

### 3.5. Catalyst Characterization

N_2_ adsorption–desorption: Each Ag/C catalyst was degassed under a nitrogen atmosphere at 300 °C for 6 h. The specific surface area of each catalyst was determined by the BET method and the pore size distribution was determined by the Barrett–Joyner–Halenda (BJH) method [44]. The adsorption–desorption analysis was performed on an ASAP2020 physical adsorption instrument (Micromeritics Instrument Corp, Norcross, GA, USA).

X-ray diffraction (XRD) was employed to evaluate the crystal structure of each catalyst. XRD patterns were obtained at 40 kV and 30 mA using Cu-Kα irradiation (λ = 0.1541 nm). Patterns were collected in the 2θ range of 10 to 80° with a step scanning rate of 10°/min.

Fourier transform infrared (FT-IR) spectroscopy was used to evaluate the functional group structure of the catalysts. Each Ag/C sample was homogeneously mixed with KBr powder and pressed into a tablet. FT-IR spectra were recorded in the range of 400–4000 cm^−1^ with a resolution of 1 cm^−1^.

X-ray photoelectron spectroscopy (XPS) analysis was performed using a sample attached to a conductive adhesive to avoid the introduction of impurities. XPS spectra were obtained using Al-Ka target radiation (hv = 1486.6 eV) with a power of 150 W, a sensitivity of 350 keps, and an analytical chamber vacuum of 10^−8^ Mbar. The fluence energies of the survey and high-resolution spectra were set to 160 eV and 40 eV, respectively. The step size of the survey spectrum was 1 eV and that of the high-resolution spectrum was 0.1 eV. Energy shift correction for the high-resolution spectrum was performed using the C ls peak (284.8 eV) of carbon impurities on the sample surface.

Thermogravimetric analysis (TGA) was used to determine the loading of AgNPs on each catalyst. Oxygen was used as a protective gas for blowing nitrogen, and the measurement was carried out at a heating rate of 10 °C/min in an air environment from 30 to 800 °C. The sample was then held at 800 °C for 120 min.

Transmission electron microscopy (TEM) was used to observe the distribution of metallic AgNPs in the composite catalyst. The composite catalyst sample was pulverized, dispersed in ethanol, and added drop by drop on a carbon grid. This grid was allowed to sufficiently dry, prior to TEM analysis. The accelerating voltage was set to 200 kV.

Scanning electron microscopy (SEM) was employed to evaluate the surface morphology of the carrier and catalyst. A small amount of the sample was directly attached to a conductive adhesive surface to avoid contamination, and the sample surface was then sprayed with gold. SEM images were obtained in high-vacuum mode with an accelerating voltage of 10 kV.

Inductively coupled plasma (ICP) was used to analyze the metal element content leached from the catalyst. ICP analysis was performed with a power of 1.20 kW, a plasma gas flow rate of 15.0 L/min, and an auxiliary gas flow rate of 1.50 L/min. The procedure was repeated three times to ensure the reliability of the results.

### 3.6. Evaluation of Catalytic Performance

Qualitative and quantitative analyses were performed using GC-MS to evaluate the performance of each catalyst. GC conditions: DB-5MS column (30 mm × 250 mm, 0.25 μm), 5% phenyl, 260 °C inlet temperature, 1 mL/min flow rate. MS conditions: 250 °C ion source temperature, electron bombardment (EI) used as the mass spectrometry ion source, scanning mode.

The conversion of the reaction was calculated by determining the integral area of the phenylacetylene before and after the reaction with a standardized volume control method. The reaction yield was calculated based on the integral area of the target product peaks as a percentage of the integral area of all substance peaks.

### 3.7. Validation of Catalyst Recoverability

The recoverability and reusability of the catalyst were studied in a recycling experiment. About 200 mg of catalyst was weighed, and alkyne substrate and halogenated reagents were utilized for the reaction according to the mass ratio reported in Section 3.4. Upon the completion of the reaction, the catalyst was filtered, collected, and dried in an oven. The dried catalyst was then calcined at 600 °C for 2 h, and the catalyst was reused in another catalytic test. After repeating this process 5 times, the mass of the catalyst was measured to calculate catalyst recovery.

## 4. Conclusions

In this study, the roots of the Rhizoma Coptidis plant were creatively used as a catalyst carrier. The distinct cell wall structure of this plant provides a large number of active sites for loading metal nanoparticles, and after carbonization, the lattice-like structure offers robust physical support for improving the stability and dispersion of the catalyst. Importantly, larger AgNPs or a higher silver content can provide more active sites, leading to enhanced catalytic activity. Consequently, the prepared carrier exhibited superb mechanical properties, porosity, stability, and surface functionality. Silver/carbon composites consisting of monodisperse AgNPs on a hierarchical mesoporous carbon carrier were successfully prepared by loading AgNPs onto the Rhizoma Coptidis roots, followed by carbonization. The prepared catalysts showed remarkable catalytic efficiency for the halogenation of a terminal alkyne, converting phenylacetylene into the corresponding halogenated derivatives with 100% conversion and high yields. Moreover, after five cycles of reuse, the Ag-3/C catalyst retained nearly 100% of its catalytic activity with more than 95% mass retention. The distinct structural characteristics and excellent activity of this catalyst are primarily responsible for its exceptional catalytic performance in the halogenation of terminal alkynes. This structure also greatly boosts the recovery performance of the catalyst, increasing its dependability and longevity in real-world applications. Notably, Rhizoma Coptidis roots are a renewable natural resource, offering an effective catalytic solution for the catalytic synthesis of terminal alkynes and their derivatives while also creating new opportunities for the development of innovative, eco-friendly catalysts.

## Figures and Tables

**Table 1:** Surface physical properties of the three Ag/C catalyst samples.

Sample	BET Surface Area (m^2^/g)	Adsorption Average Pore Diameter (nm)	Pore Volume (cm^3^/g)
AG-1/C	83.99	10.61	0.22
AG-2/C	115.92	8.07	0.23
AG-3/C	176.90	7.26	0.32

**Table 2:** Conversion and yield (determined by GC-MS) for the halogenation of phenylacetylene by Ag/C catalysts.

Entry	Catalyst	Conversion/%	Yield/%
a ^1^	AG-1/C	100	57.6
a	AG-2/C	100	86.7
a	AG-3/C	100	89.4
b ^2^	AG-1/C	100	62.6
b	AG-2/C	100	92.3
b	AG-3/C	100	96.4
c ^3^	AG-1/C	100	60.3
c	AG-2/C	100	93.8
c	AG-3/C	100	97.4

Note ^1^: “a” refers to the chlorination of phenylacetylene; ^2^: “b” refers to the bromination of phenylacetylene; ^3^: “c” refers to the iodination of phenylacetylene.

## Data Availability

The data that support the findings of this study are available from the corresponding author upon reasonable request.

## Supplementary Materials

The following supporting information can be downloaded at https://www.mdpi.com/article/10.3390/molecules30030567/s1: The GC profiles for the halogenation reactions catalyzed by AG-1/C, AG-2/C, and AG-3/C are presented in the Supplementary Information, Figure S1: (a) GC spectrum of AG-1/C bromination experiment, (b) GC spectrum of AG-2/C bromination experiment, (c) GC spectrum of AG-3/C bromination experiment, (d) GC spectrum of AG-1/C chlorination experiment, (e) GC spectrum of AG-2/C chlorination experiment, (f) GC spectrum of AG-3/C chlorination experiment, (g) GC spectrum of AG-1/C iodination experiment, (h) GC spectrum of AG-2/C iodination experiment, (i) GC spectrum of AG-3/C iodination experiment.
