# Supplementary material for: Coptis Root-Derived Hierarchical Carbon-Supported Ag Nanoparticles for Efficient and Recyclable Alkyne Halogenation
Source: Molecules. 2025 Jan 26;30(3):567. doi: 10.3390/molecules30030567 (PMC11820834; doi:10.3390/molecules30030567)
Supplement: Supplementary file 1 [file molecules-30-00567-s001.zip › molecules-3402011-supplementary.pdf]

a

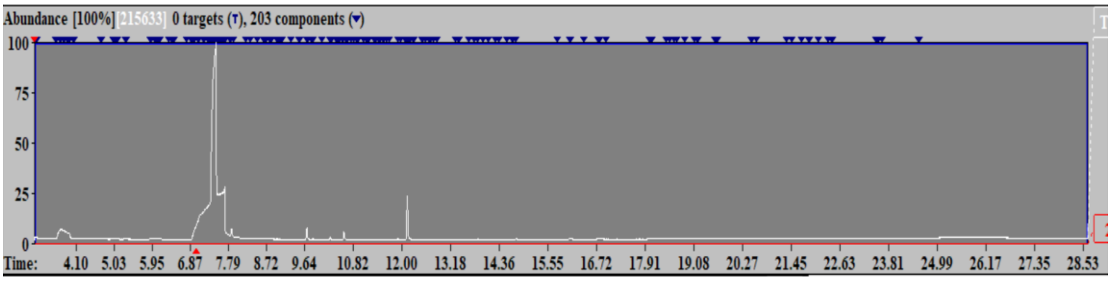
Abundance [100%] [215633] 0 targets (T), 203 components (▼)
100
75
50
25
0
Time: 4.10 5.03 5.95 6.87 7.79 8.72 9.64 10.82 12.00 13.18 14.36 15.55 16.72 17.91 19.08 20.27 21.45 22.63 23.81 24.99 26.17 27.35 28.53

b

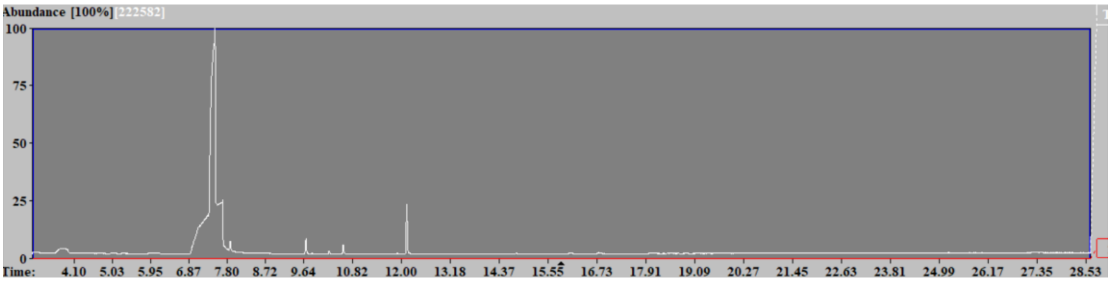
Abundance [100%] [222582]
100
75
50
25
0
Time: 4.10 5.03 5.95 6.87 7.80 8.72 9.64 10.82 12.00 13.18 14.37 15.55 16.73 17.91 19.09 20.27 21.45 22.63 23.81 24.99 26.17 27.35 28.53

c

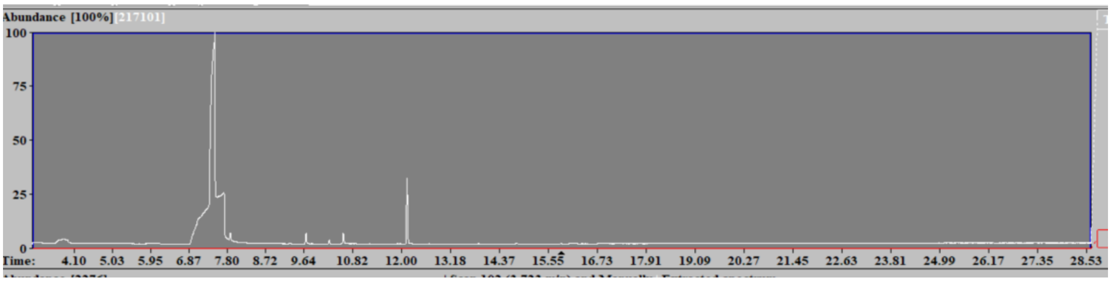
Abundance [100%] [217101]
100
75
50
25
0
Time: 4.10 5.03 5.95 6.87 7.80 8.72 9.64 10.82 12.00 13.18 14.37 15.55 16.73 17.91 19.09 20.27 21.45 22.63 23.81 24.99 26.17 27.35 28.53

d

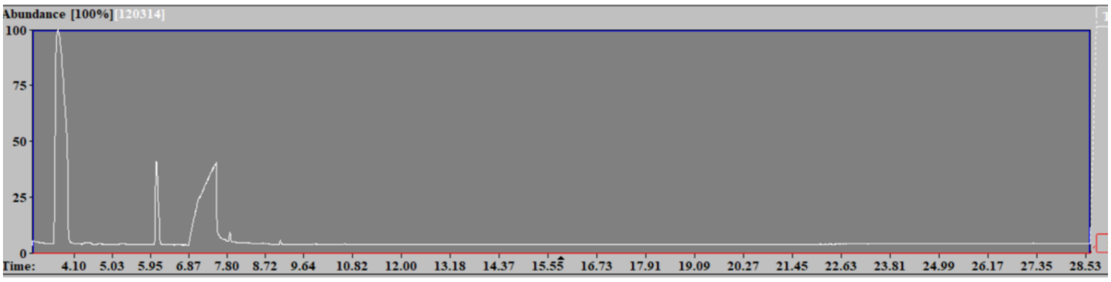
Abundance [100%] [120314]
100
75
50
25
0
Time: 4.10 5.03 5.95 6.87 7.80 8.72 9.64 10.82 12.00 13.18 14.37 15.55 16.73 17.91 19.09 20.27 21.45 22.63 23.81 24.99 26.17 27.35 28.53

e

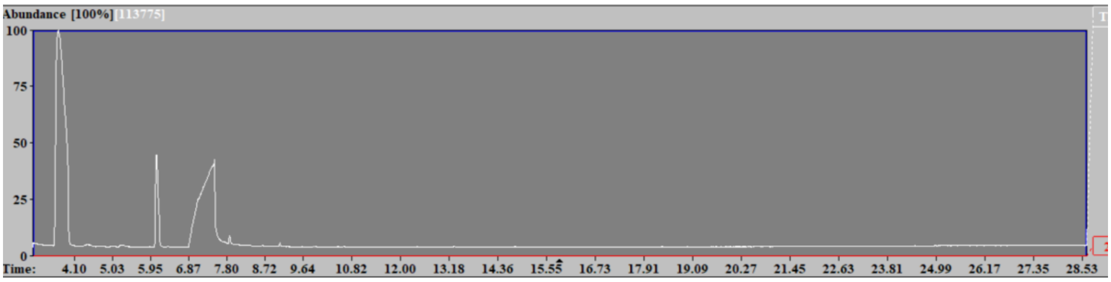
Abundance [100%] [113775]
100
75
50
25
0
Time: 4.10 5.03 5.95 6.87 7.80 8.72 9.64 10.82 12.00 13.18 14.36 15.55 16.73 17.91 19.09 20.27 21.45 22.63 23.81 24.99 26.17 27.35 28.53

f

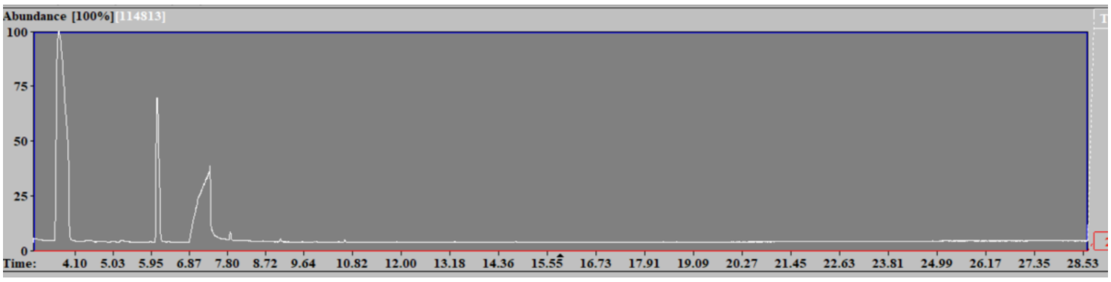

g

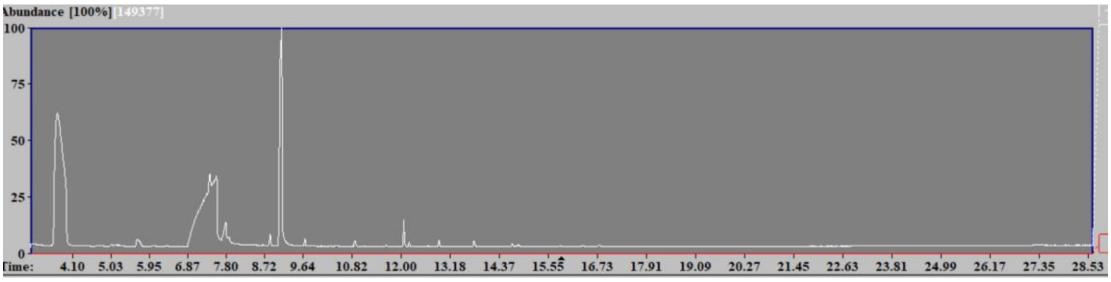

h

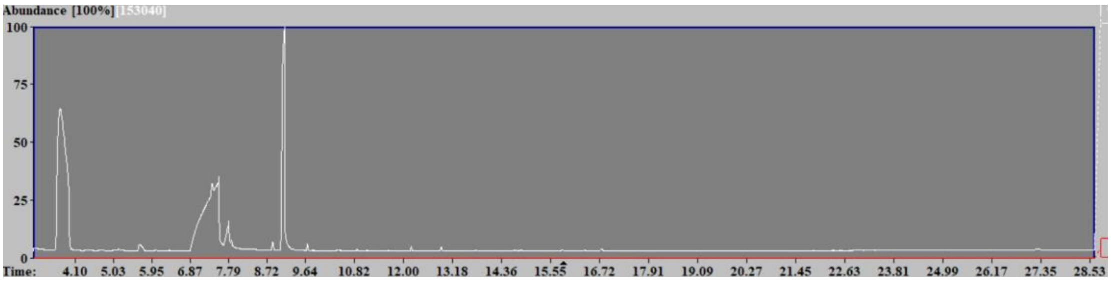

i

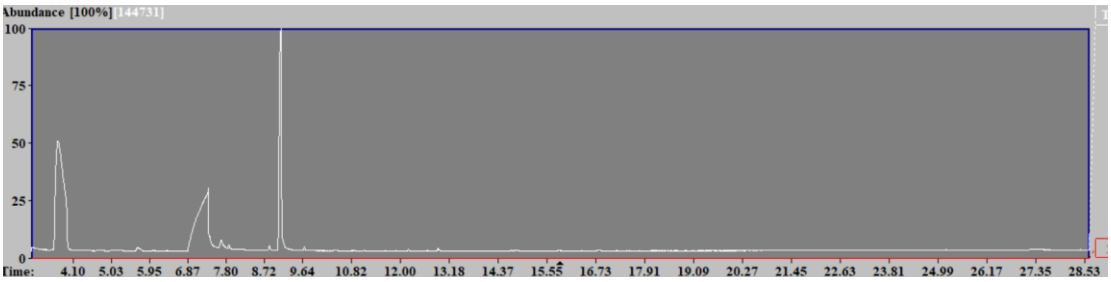

Supplementary Figure S1. (a) GC spectrum of AG-1/C bromination experiment, (b) GC spectrum of AG-2/C bromination experiment, (c) GC spectrum of AG-3/C bromination experiment, (d) GC spectrum of AG-1/C chlorination experiment, (e) GC spectrum of AG-2/C chlorination experiment, (f) GC spectrum of AG-3/C chlorination experiment, (g) GC spectrum of AG-1/ C iodination experiment, (h) GC spectrum of AG-2/C iodination experiment, (i) GC spectrum of AG-3/C iodination experiment
